# Supplementary figures and images for: The amount of Nck rather than N-WASP correlates with the rate of actin-based motility of Vaccinia virus
Source: Microbiol Spectr. 2023 Oct 19;11(6):e01529-23. doi: 10.1128/spectrum.01529-23 (PMC10883800; doi:10.1128/spectrum.01529-23)

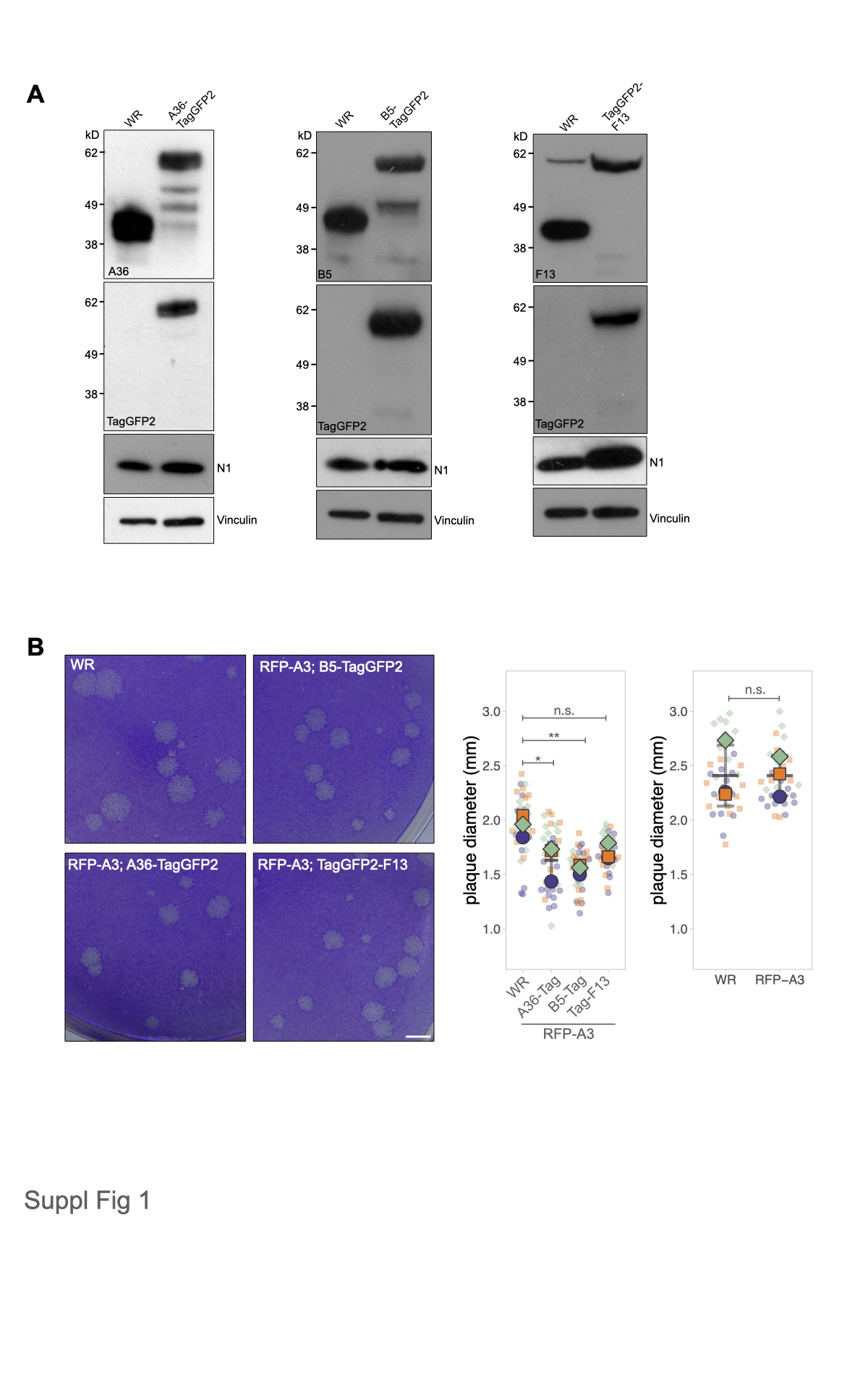

Supplement: Fig. S1 — Validation of TagGFP2-labeled recombinant viruses. [file spectrum.01529-23-s0001.tif]

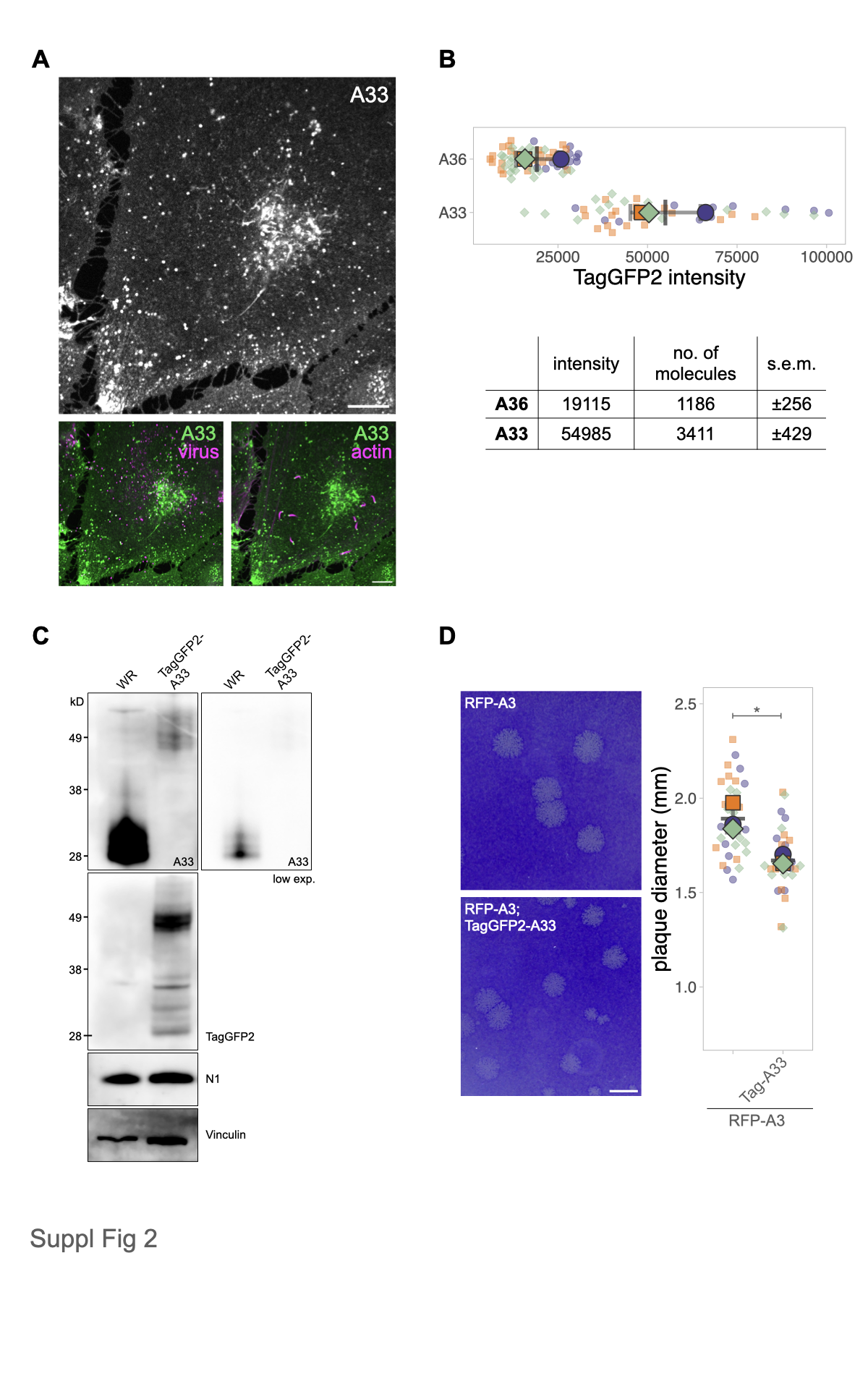

Supplement: Fig. S2 — Validation of TagGFP2-A33 recombinant virus. [file spectrum.01529-23-s0002.tif]

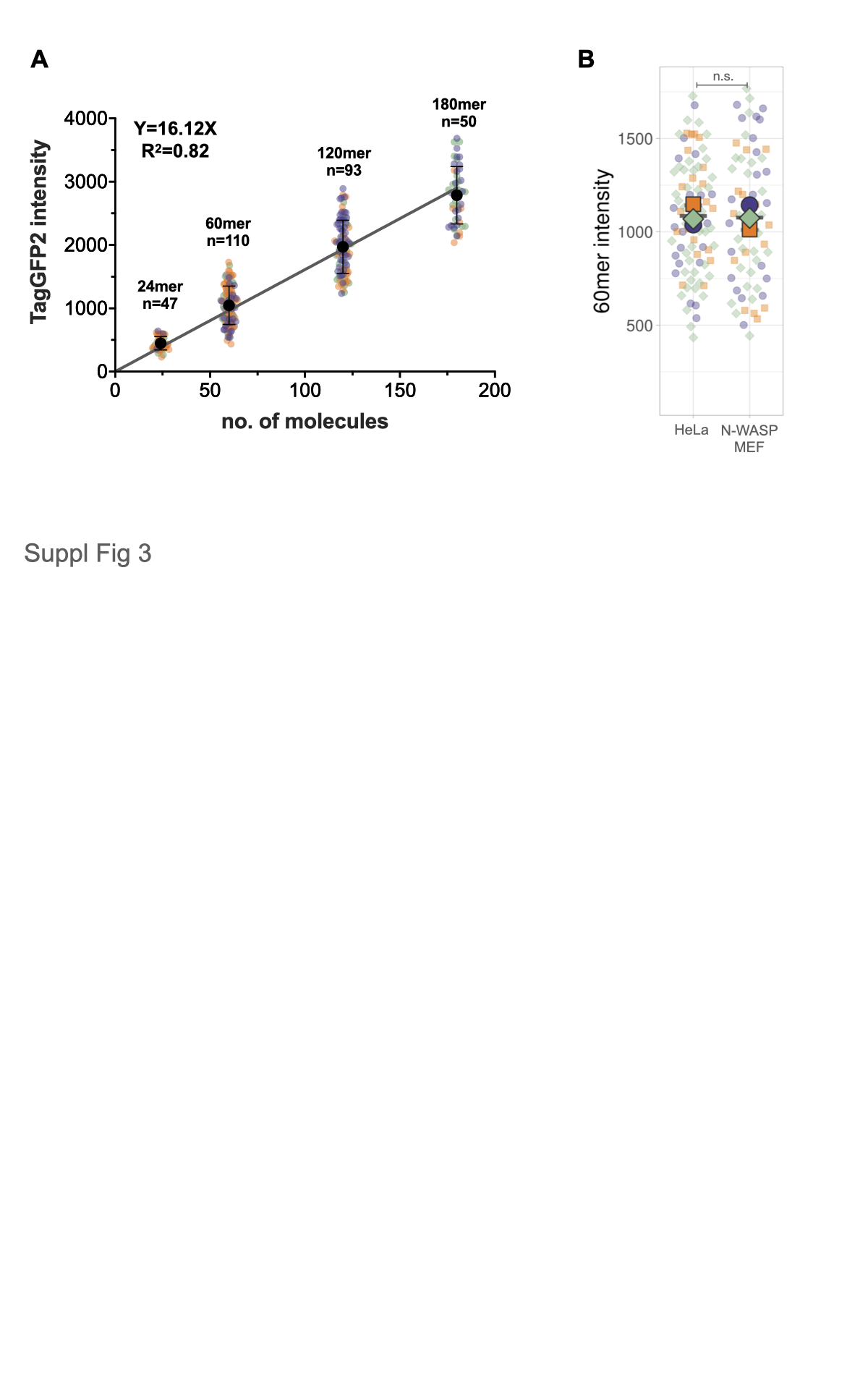

Supplement: Fig. S3 — Validation of fluorescent nanocages. [file spectrum.01529-23-s0003.tif]

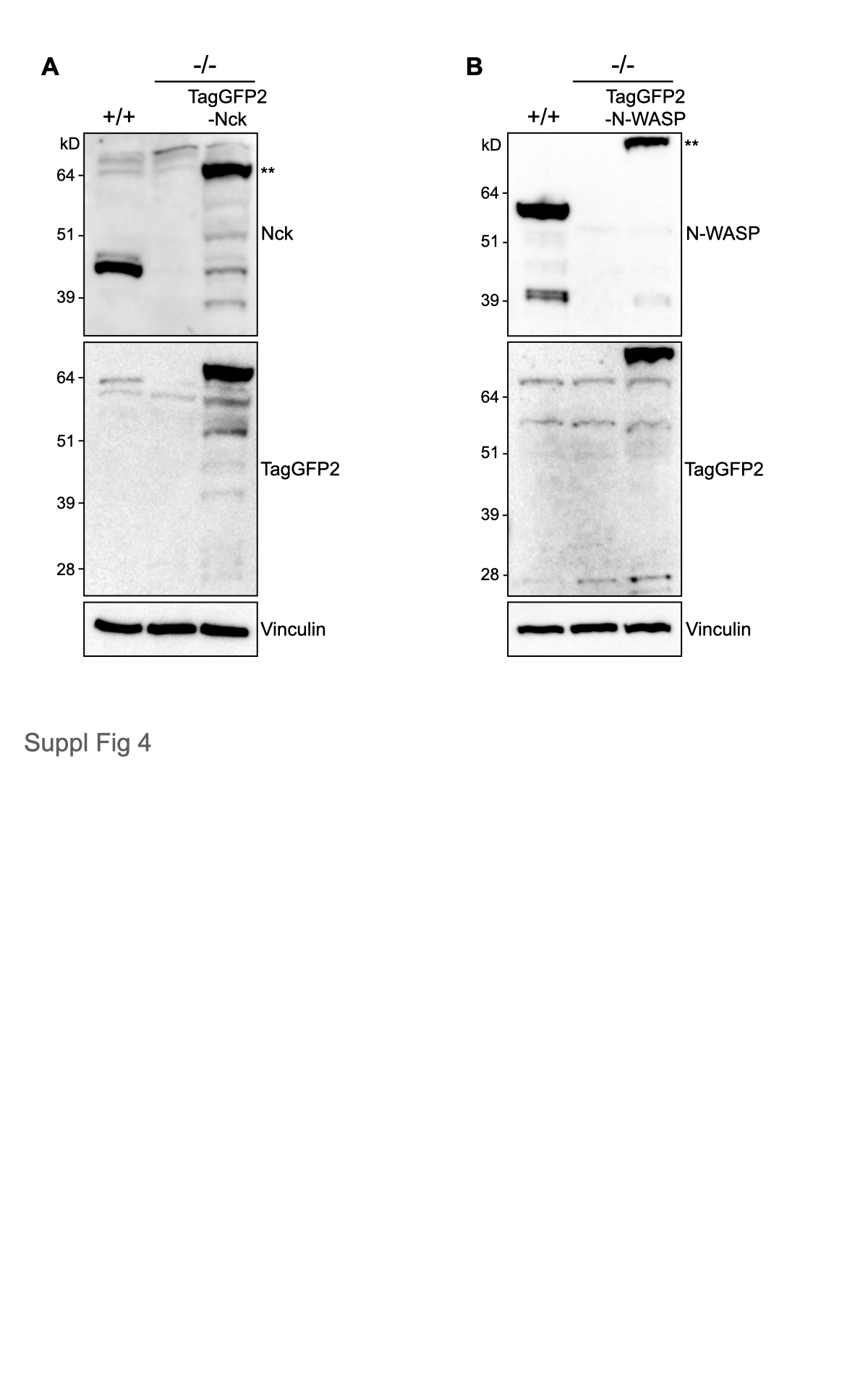

Supplement: Fig. S4 — Validation of stable cell lines expressing TagGFP2-Nck and TagGFP2-N-WASP. [file spectrum.01529-23-s0004.tif]
