# Supplementary material for: The amount of Nck rather than N-WASP correlates with the rate of actin-based motility of Vaccinia virus
Source: Microbiol Spectr. 2023 Oct 19;11(6):e01529-23. doi: 10.1128/spectrum.01529-23 (PMC10883800; doi:10.1128/spectrum.01529-23)
Supplement: Supplemental legends — Legends for Fig. S1 to S4. [file spectrum.01529-23-s0005.docx]

**SUPPLEMENTAL MATERIAL**

These four supplemental figures provide validation of reagents (recombinant viruses and cell lines) and of the quantitative imaging technique used in this study. Supplement 2 also provides data on the number of A33 molecules accumulating on virus particles.

**Supplement 1: Validation of TagGFP2-labelled recombinant viruses**

**A.** Immunoblot analyses of total cell lysates from HeLa cells infected with Vaccinia WR or the indicated TagGFP2 fusion viruses at 9 hours post-infection. **B.** Representative images of plaques produced by the indicated viruses in confluent BS-C-1 cells at 72 hours post-infection. Scale bar = 2 mm**.** The graph shows quantification of plaque diameter of the recombinant viruses compared with WR. The graph on the right shows that the RFP-A3 tag that labels the virus core does not confer a change in plaque diameter. All error bars represent S.D and the distribution of data from each experiment is shown using a “SuperPlot”. A total of 36 plaques per virus were measured over three independent experiments. Dunnett’s multiple comparison’s test (left graph) or Welch’s t test (right graph) was used to determine statistical significance; ns, p >0.05; * p ≤ 0.05; ** p ≤ 0.01.

**Supplement 2: Validation of TagGFP2-A33 recombinant virus and determination of A33 numbers at CEV**

**A.** Representative single-plane confocal images of live HeLa cell infected with a recombinant virus expressing TagGFP2-A33 at its endogenous locus. Actin is labelled with LifeAct-iRFP, and all virus particles are labelled with RFP-A3. Images are acquired at 9 hours post-infection. Scale bar = 10 μm. **B.** The graph shows quantification of background-subtracted raw integrated TagGFP2 intensities at actin-polymerising virions. All error bars represent S.D and the distribution of data from each experiment is shown using a “SuperPlot”. A total of 46-59 particles were measured per recombinant virus over three independent experiments. The table shows the calculated number of A36 and A33 molecules at actin-polymerising virus particles in HeLa cells (s.e.m. = standard error of mean). **C.** Immunoblot analyses of total cell lysates from HeLa cells infected with Vaccinia WR or the TagGFP2-A33 fusion virus at 9 hours post-infection. **D.** Representative images of plaques produced by the indicated viruses in confluent BS-C-1 cells at 72 hours post-infection. Scale bar = 2 mm**.** The graph shows quantification of plaque diameter of the recombinant virus compared with a virus expressing the RFP-A3 tag alone. All error bars represent S.D and the distribution of data from each experiment is shown using a “SuperPlot”. A total of 36 plaques per virus were measured over three independent experiments. Welch’s t test was used to determine statistical significance; * p ≤ 0.05.

**Supplement 3: Validation of fluorescent nanocages**

**A.** The graph shows quantification of background-subtracted raw integrated fluorescence intensities of nanocages transiently expressed in live HeLa cells treated with 500 nM AP21967. Linear line of regression is fitted to the data. A total of 47-110 membrane-tethered nanocages were measured per condition over three independent experiments. **B.** Quantification of background-subtracted raw integrated intensity of 60mer TagGFP2-nanocages transiently expressed in HeLa cells or N-WASP+/+ parental MEFs treated with 500 nM AP21967. A total of 66-84 membrane-tethered spots were measured over three independent experiments. All error bars represent S.D and the distribution of data from each experiment is shown using a “SuperPlot”. Welch’s t test (panel B) was used to determine statistical significance; ns, p >0.05.

**Supplement 4: Validation of stable cell lines expressing TagGFP2-Nck and TagGFP2-N-WASP**

**A.** Immunoblot analyses of total cell lysates from Nck1/Nck2 null MEFs with and without stable expression of TagGFP2-Nck together with parental wild-type cells. **B.** Immunoblot analyses of total cell lysates from N-WASP null MEFs with and without stable expression of TagGFP2-N-WASP together with parental wild-type cells. Immunoblot analyses was performed with the indicated antibodies. ** indicates the band corresponding to the TagGFP2-labelled protein.
